# Supplementary material for: Effective Circulating Tumor Cell Isolation Using Epithelial and Mesenchymal Markers in Prostate and Pancreatic Cancer Patients
Source: Cancers (Basel). 2023 May 18;15(10):2825. doi: 10.3390/cancers15102825 (PMC10216737; doi:10.3390/cancers15102825)
Supplement: Supplementary file 1 [file cancers-15-02825-s001.zip › cancers-2355409-supplementary.pdf]

Supplementary Materials

Effective Circulating Tumor Cell Isolation Using Epithelial and Mesenchymal Markers in Prostate and Pancreatic Cancer Patients

Jiwon Cha [1], Hyungseok Cho [1], Jae-Seung Chung [2], Joon Seong Park [3] and Ki-Ho Han [1,*]

[1] Department of Nanoscience and Engineering, Center for Nano Manufacturing, Inje University, Gimhae 50834, Republic of Korea; ckjw51@gmail.com (J.C.); elshaddai88@naver.com (H.C.)
[2] Department of Urology, Haeundae Paik Hospital, Inje University, Busan 48108, Republic of Korea; biogen@hanmail.net
[3] Pancreatobiliary Cancer Clinic, Department of Surgery, Gangnam Severance Hospital, Yonsei University College of Medicine, Seoul 06229, Republic of Korea; jspark330@yuhs.ac
* Correspondence: mems@inje.ac.kr; Tel.: +82-(55)-3203715; Fax: +82-(55)-3203631

Table S1. Information of five healthy donor, a patient with benign prostatic hyperplasia (BPH), and seventeen patients with prostate cancer (to be continued)

Patient ID	Gender	Age	Stage	Therapy	Isolation antibody	CTCs /mL	WBCs /mL
H1	M	51			EpCAM	0	691
					Vimentin	0	698
					Ep. + Vim.	0	1004
H2	M	68			EpCAM	0	1262
					Vimentin	0	1268
					Ep. + Vim.	0	1113
H3	M	77			EpCAM	0	1151
					Vimentin	0	1333
					Ep. + Vim.	0	914
H4	M	51			EpCAM	0	1031
					Vimentin	0	1137
					Ep. + Vim.	0.3	998
H5	M	53			EpCAM	0	683
					Vimentin	0	1496
					Ep. + Vim.	1	1496
BPH	M	68			EpCAM	0	280
					Vimentin	0.7	514
					Ep. + Vim.	0.7	308
Ps1	M	67	T2	Prostatectomy	EpCAM	4	1362
					Vimentin	1	1830
					Ep. + Vim.	5	1089
Ps2	M	67	T2	Prostatectomy	EpCAM	5	2590
					Vimentin	1	2078
					Ep. + Vim.	6	1555
Ps3	M	79	T2	Prostatectomy	EpCAM	9.2	1895
					Vimentin	2.4	1636
					Ep. + Vim.	11.2	1569
Ps4	M	69	T2	Prostatectomy	EpCAM	10	1106
					Vimentin	2	1292
					Ep. + Vim.	13	917
Ps5	M	60	T3	Prostatectomy	EpCAM	4	1316
					Vimentin	6	1548
					Ep. + Vim.	12	1257

Table S1. (Continued)

Patient ID	Gender	Age	Stage	Therapy	Isolation antibody	CTCs /mL	WBCs /mL
Ps6	M	79	T4	Radiation	EpCAM	7.3	1636
					Vimentin	6.7	1607
					Ep. + Vim.	12.7	1927
Ps7	M	78	mHSPC	Abiraterone	EpCAM	8	1804
					Vimentin	5	1080
					Ep. + Vim.	14	898
Ps8	M	75	mHSPC	ADT	EpCAM	3	1796
					Vimentin	12	1380
					Ep. + Vim.	18	901
Ps9	M	64	mHSPC	ADT	EpCAM	10.7	2610
					Vimentin	12.3	2638
					Ep. + Vim.	23.7	2177
Ps10	M	65	mHSPC	ADT	EpCAM	16.7	3157
					Vimentin	19.3	1947
					Ep. + Vim.	36	2423
Ps11	M	73	mHSPC	Abiraterone	EpCAM	21.5	2354
					Vimentin	8	3072
					Ep. + Vim.	43	2949
Ps12	M	72	mCRPC	Docetaxel	EpCAM	5	1722
					Vimentin	5	1535
					Ep. + Vim.	11	1684
Ps13	M	81	mCRPC	Xtandi	EpCAM	10	1098
					Vimentin	10.5	1047
					Ep. + Vim.	25.5	1173
Ps14	M	75	mCRPC	Docetaxel	EpCAM	13.3	3168
					Vimentin	13.3	2160
					Ep. + Vim.	34	2059
Ps15	M	65	mCRPC	Abiraterone	EpCAM	21	1145
					Vimentin	22	836
					Ep. + Vim.	36	828
Ps16	M	77	mCRPC	Docetaxel	EpCAM	29.3	1526
					Vimentin	6.7	1496
					Ep. + Vim.	52	1496
Ps17	M	72	mCRPC	Docetaxel	EpCAM	48	5420
					Vimentin	56	2250
					Ep. + Vim.	122	2600

Table S2. Information of five patients with pancreatic cancer

Patient ID	Gender	Age	Stage	Therapy	Isolation antibody	CTCs /mL	WBCs /mL
Pan1	M	64	III	Chemotherapy	EpCAM	0.7	2098
					Vimentin	2	2036
					Ep. + Vim.	2.7	2178
Pan2	M	64	II	Chemotherapy	EpCAM	1.3	387
					Vimentin	6	322
					Ep. + Vim.	6	278
Pan3	M	72	I	Surgery	EpCAM	3.5	4575
					Vimentin	2	3920
					Ep. + Vim.	7	3100
Pan4	F	75	III	Surgery	EpCAM	0	1810
					Vimentin	4	1224
					Ep. + Vim.	16	714
Pan5	M	68	I	Surgery	EpCAM	2	404
					Vimentin	3.2	287
					Ep. + Vim.	22.4	218

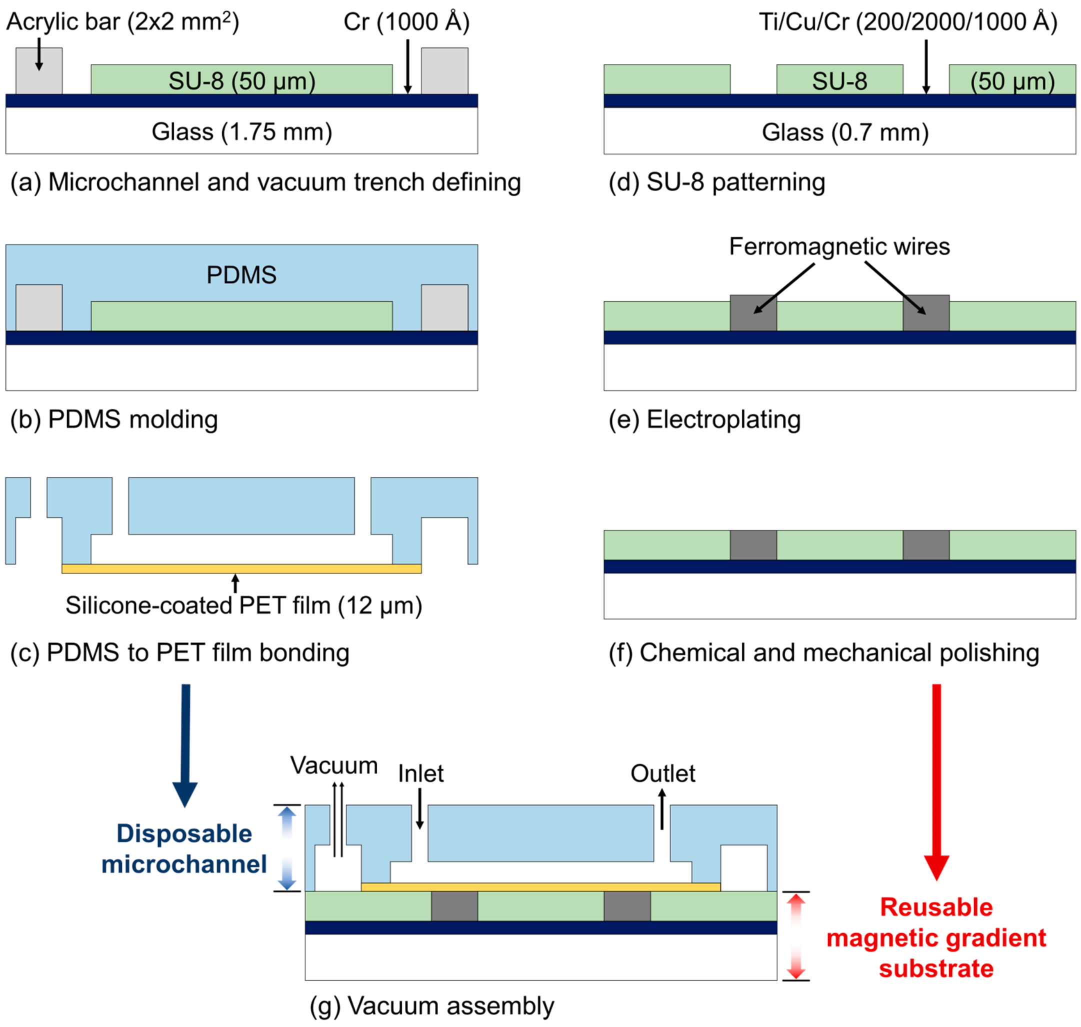

Figure S1. Fabrication process of CTC-dChip. (a) Depositing 1000 Å of Cr on a glass slide, followed by patterning SU-8 (3050, MicroChem Co.) to define the 50 μm-height microchannel, and using ultraviolet adhesive to bond an acrylic square bar (2 × 2 mm^2) for outlining the vacuum trench. (b) Creating a PDMS mold to produce the microstructured PDMS replica. (c) Employing a 1.5 mm diameter punch to form inlet and outlet reservoirs, as well as a vacuum hole. Utilizing oxygen plasma to bond the PDMS replica and the 12-μm-thick silicone-coated release PET film, resulting in the disposable microchannel. (d) Pattering a 50 μm-thick micromold with SU-8 on a Ti/Cu/Cr (200/2000/1000 Å) deposited glass substrate. (e) Fabricating ferromagnetic wires through electroplating, and (f) subsequently flattening them using chemical and mechanical polishing. (g) Assembling the disposable microchannel and the reusable magnetic gradient substrate under vacuum conditions to produce the CTC-dChip.

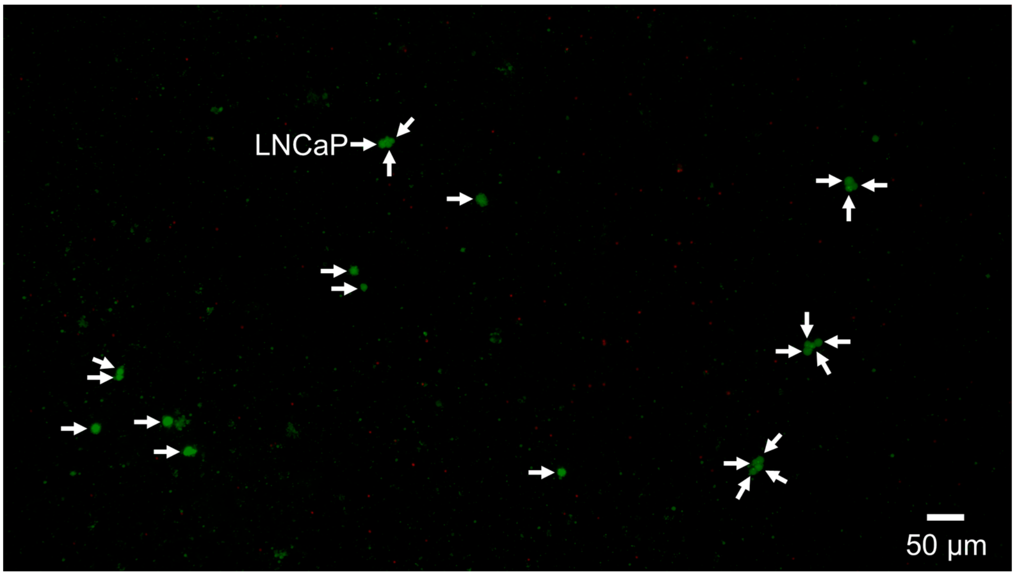

(a)

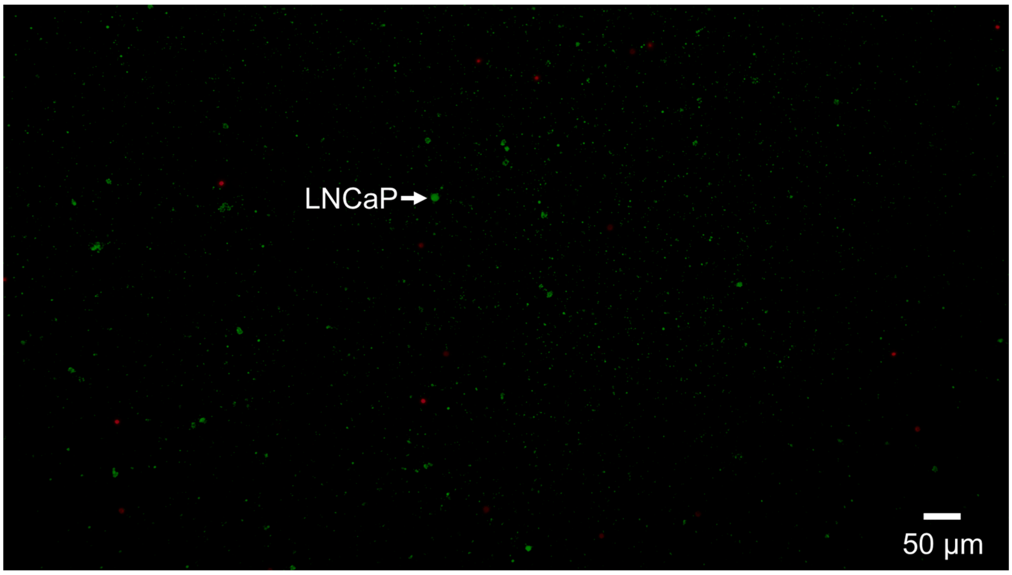

(b)

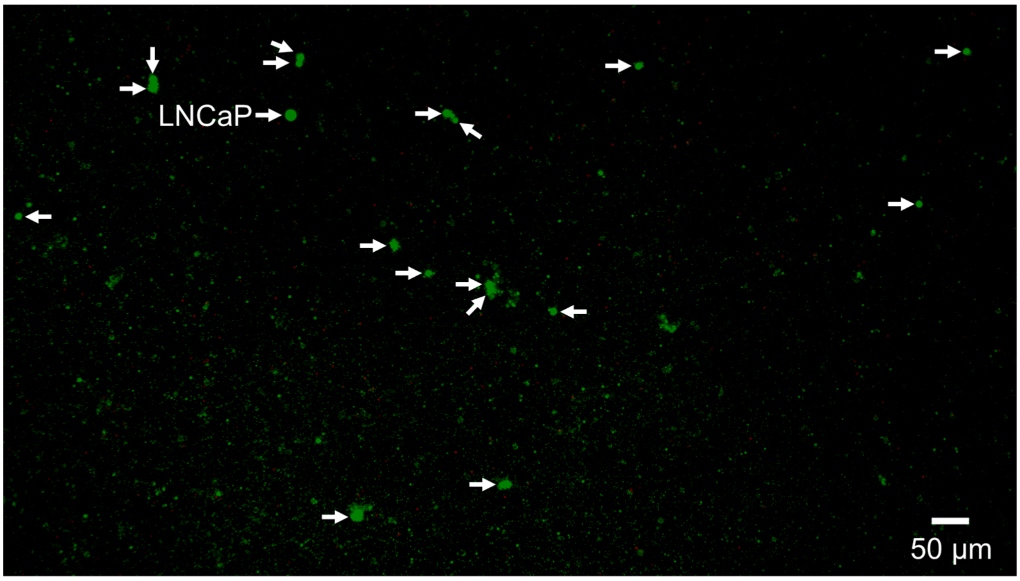

(c)

Figure S2. Fluorescence-based live/dead assay was conducted for LNCaP cells isolated using the CTC-dChip with (a) EpCAM antibodies alone, (b) vimentin antibodies alone, and (c) both combined. For the live/dead assay, 200 LNCaP cells were spiked into 3 mL of healthy blood. Among the LNCaP cells isolated with EpCAM antibodies alone, there were 195 live cells and 1 dead cell. When using vimentin antibodies alone, there were 8 live cells and 0 dead cells, while in the case of using vimentin antibodies alone, there were 197 live cells and 2 dead cells.

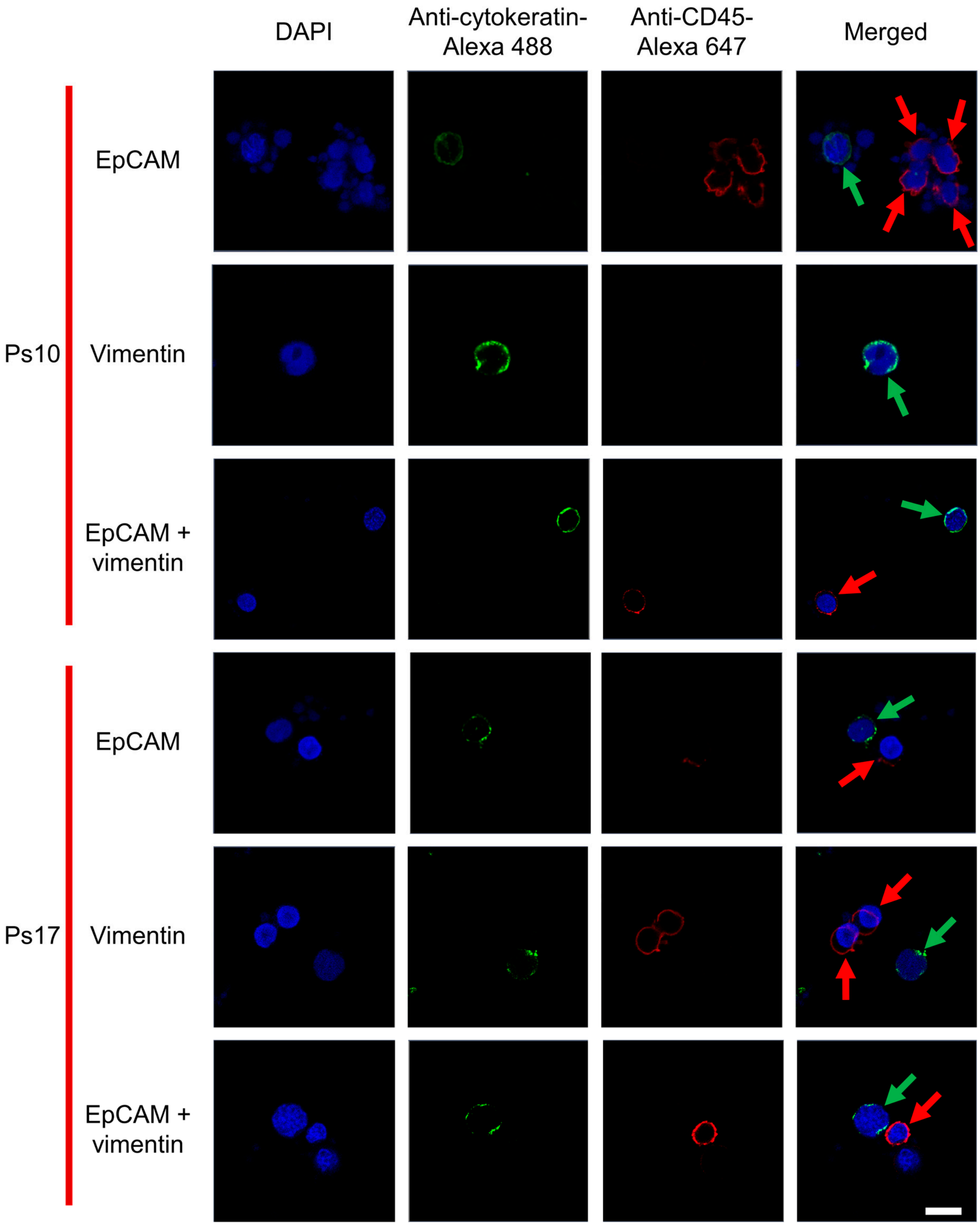

Figure S3. Photomicrographs of CTCs (indicated by green arrows; ➡) and WBCs (marked by red arrows; ➡), isolated from patients (Ps10 and Ps17) with prostate cancer and stained using immunofluorescence dyes: DAPI for nuclei, anti-cytokeratin-Alexa 488 for CTCs, and anti-CD45-Alexa 647 for WBCs. The scale bar represents 10 μm.

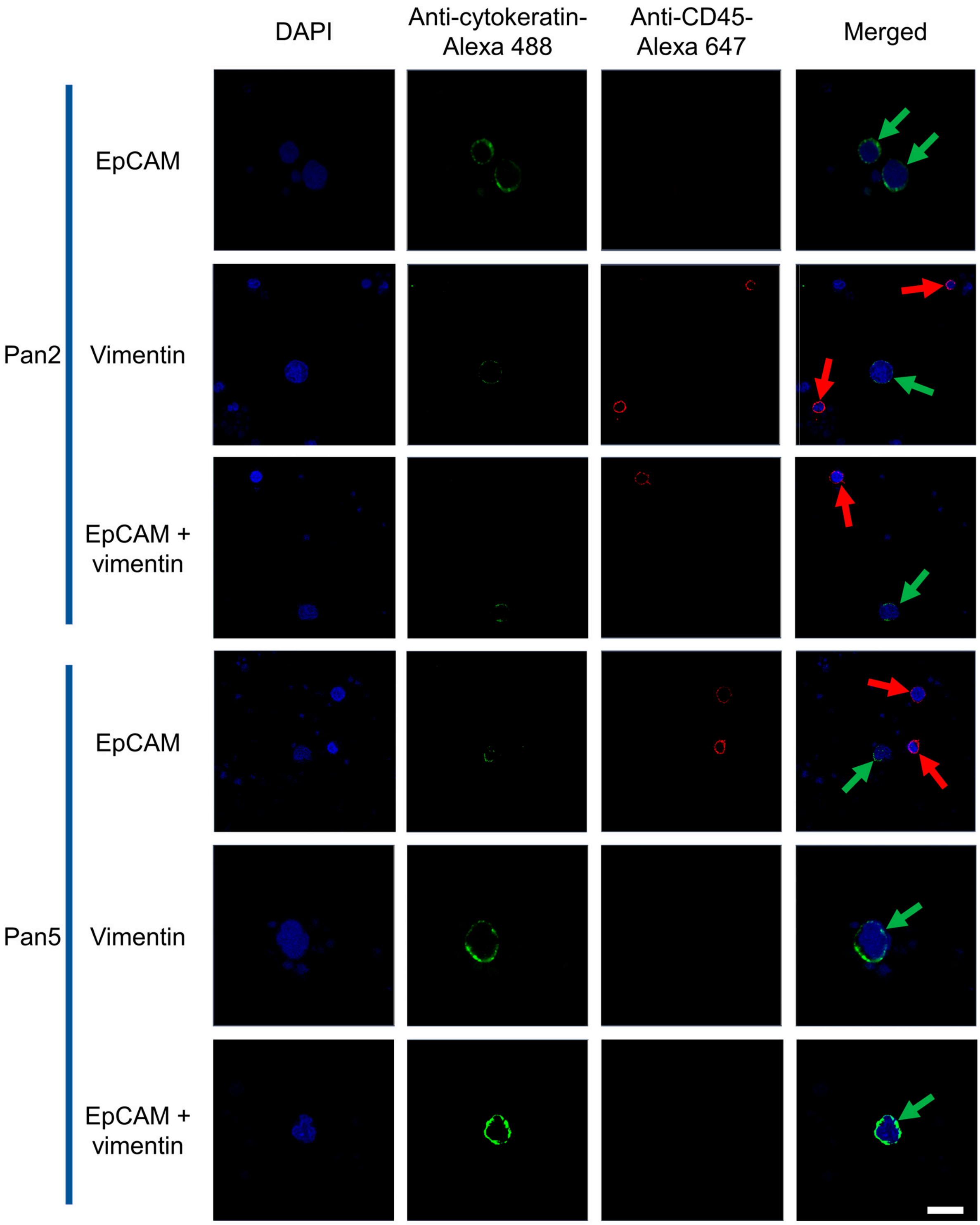

Figure S4. Photomicrographs of CTCs (indicated by green arrows; ➡) and WBCs (marked by red arrows; ➡), isolated from patients (Pan2 and Pan5) with pancreatic cancer and stained using immunofluorescence dyes: DAPI for nuclei, anti-cytokeratin-Alexa 488 for CTCs, and anti-CD45-Alexa 647 for WBCs. The scale bar represents 10 μm.
